# The complete mitochondrial genome of *Toxotes chatareus* (Toxotes; Toxotidae; Carangaria) assembled by the next-generation sequencing data and phylogenetic analysis of Carangaria

**DOI:** 10.1080/23802359.2021.1991246

**Published:** 2021-10-15

**Authors:** Liang Zhu, Hui Jiang, Longlong Zhang, Jingmei Cha, Bingyu Mao, Yongxin Li

**Affiliations:** a State Key Laboratory of Genetic Resources and Evolution, Kunming Institute of Zoology, Chinese Academy of Sciences, Kunming, China; b Kunming College of Life Science, University of Chinese Academy of Sciences, Kunming, China; c College of Life Science, Hainan Normal University, Haikou, China; d State Key Laboratory for Conservation and Utilization of Bio-Resources and Key Laboratory for Microbial Resources of the Ministry of Education, School of Life Sciences, Yunnan University, Kunming, China; e School of Ecology and Environment, Northwestern Polytechnical University, Xi’an, China

**Keywords:** *Toxotes chatareus*, mitochondrial genome, next-generation sequencing, phylogeny

## Abstract

We present the complete mitochondrial genome of *Toxotes chatareus* yielded by the next-generation sequencing data in this study. The complete mitochondrial genome of *T. chatareus* has 16,543 bp and contained 13 protein-coding genes, 22 transfer RNA genes, two ribosomal RNA genes, and a single control region (D-loop). The overall base composition was A 28.75%, C 29.80%, G 15.77%, T 25.68% and its gene arrangement was similar with other Carangaria mitochondrial genomes. Additionally, the phylogenetic relationships of 13 Carangaria species based on the complete mitochondrial genome was analyzed using the neighbor-joining method. The result showed *T. chatareus* was clustered with *L. lactarius* suggesting the close phylogenetic affinity they owned. Together, the complete mitochondrial genome of *T. chatareus* would be beneficial for the study of phylogenetic relationship, taxonomic classification and phylogeography of the Carangaria.

*Toxotes chatareus* belongs to Toxotes; Toxotidae; Carangaria (Allen 1978, 2004; Simon et al. 2010; Schoch et al. 2020; Froese and Pauly 2021), is mainly distributed in the brackish water of mangrove-lined estuaries along the South Pacific and Indian Oceans, although it can also be found far upstream in fresh waters and more saline coastal waters (Allen 2001; Temple 2007; Temple et al. 2010). It is well known for its ability to shoot down insects with a jet of water squirted from the mouth (Elshoud and Koomen 1985; Simon et al. 2009, 2012). Several researches have focused on the mechanism of spitting (Timmermans 2001), visual capabilities (Temple et al. 2013) and brain atlas (Karoubi et al. 2016). Yet, little information about its genetic characteristics is available. In order to find new DNA markers for the future study of the phylogenetic relationship, taxonomic classification and phylogeography of the Carangaria, we assembled and acquired the complete mitogenome of *T. chatareus* by the next-generation sequencing (NGS) data.

The *T. chatareus* sample was acquired from Thailand (14”N; 100”E) and stored in a refrigerator of −80 °C at School of Ecology and Environment, Northwestern Polytechnical University underthe voucher number 20190815AP01. The species was identified based on morphologic features and *COI* gene (SequenceID: AP006806.1). The NGS data used in this study was produced by our previous study (Lü et al. 2021) and could be acquired from the public database NCBI with the accession number SRX8345786. The short reads which were produced from the HiSeq platform were used to assemble the mitogenome by MitoZ software with default parameters (Meng et al. 2019). The MITOS Web Server (Bernt et al. 2013) and tRNAscan-SE Search Server (Chan & Lowe 2019) were used to predict and annotate the mitogenome. Whole mitogenome sequence of *T. chatareus* was 16,543 bp in length, containing 13 protein-coding genes, 22 tRNA genes, two rRNA genes, and one control region (D-loop). The gene arrangement and base content were similar with other Carangaria species (Lv et al. 2016; Wang et al. 2016; Gan et al. 2017; Yang et al. 2019; Tabassum et al. 2020). Almost all the protein-coding genes were encoded by H-strand with the exception of *ND6* and eight tRNAs (Gln, Ala, Asn, Cys, Tyr, Ser, Glu, Pro) genes located on L-strand. The base composition was A 28.75%, C 29.80%, G 15.77%, T 25.68%. AT and GC contents were 54.43% and 45.57%, respectively. Twelve protein-coding genes started with an ATG initiation codon, while *COX1* used GTG as an initiation codon. For the termination codon, eight protein-coding genes (*ATP6*, *ATP8*, *COX1*, *COX3*, *ND1*, *ND2*, *ND4L*, and *ND5*) ended with TAA, two protein-coding genes (*ND3* and *ND6*) with TAG, and *COX2*, *CYTB* and *ND4* have an incomplete stop codon T–. The 13 protein-coding genes were 11,433 bp in length, accounting for 69.11% of the whole mitogenome, which encodes 3,811 amino acids in total. The lengths of 12S rRNA located between tRNA^Phe^ and tRNA^Val^ and 16S rRNA located between tRNA^Val^ and tRNA^Leu^ were 959 bp and 1,697 bp, respectively. The control region (D-Loop) typically located between tRNA^Pro^ and tRNA^Phe^, was 828 bp in length.

To further investigate the phylogenetic location of *T. chatareus* in Carangaria, the phylogenetic relationship was constructed in this study. Specifically, we first downloaded the mitochondrial genome of 13 species (including 12 Carangaria species and *Danio rerio*) in NCBI database. Then, the sequence alignment of these 14 species (including *T. chatareus*) was conducted by multiple sequence alignment program ClustalW (Thompson et al. 1994) in BioEdit software (Hall 1999). At last, the phylogenetic tree was constructed using the neighbor joining (NJ) method with 10,000 bootstrap replications using MEGA7 (Kumar et al. 2016). The result of the phylogeny shows that *T. chatareus* is clustered with the *L. lactarius* (Figure 1), which is belongs to Lactariidae, suggesting close phylogenetic relationship they owned. We expect that the information of the complete mitogenome of *T. chatareus* would be beneficial for the study of phylogenetic relationship, taxonomic classification and phylogeography of the Carangaria in the future.

**Figure 1.**
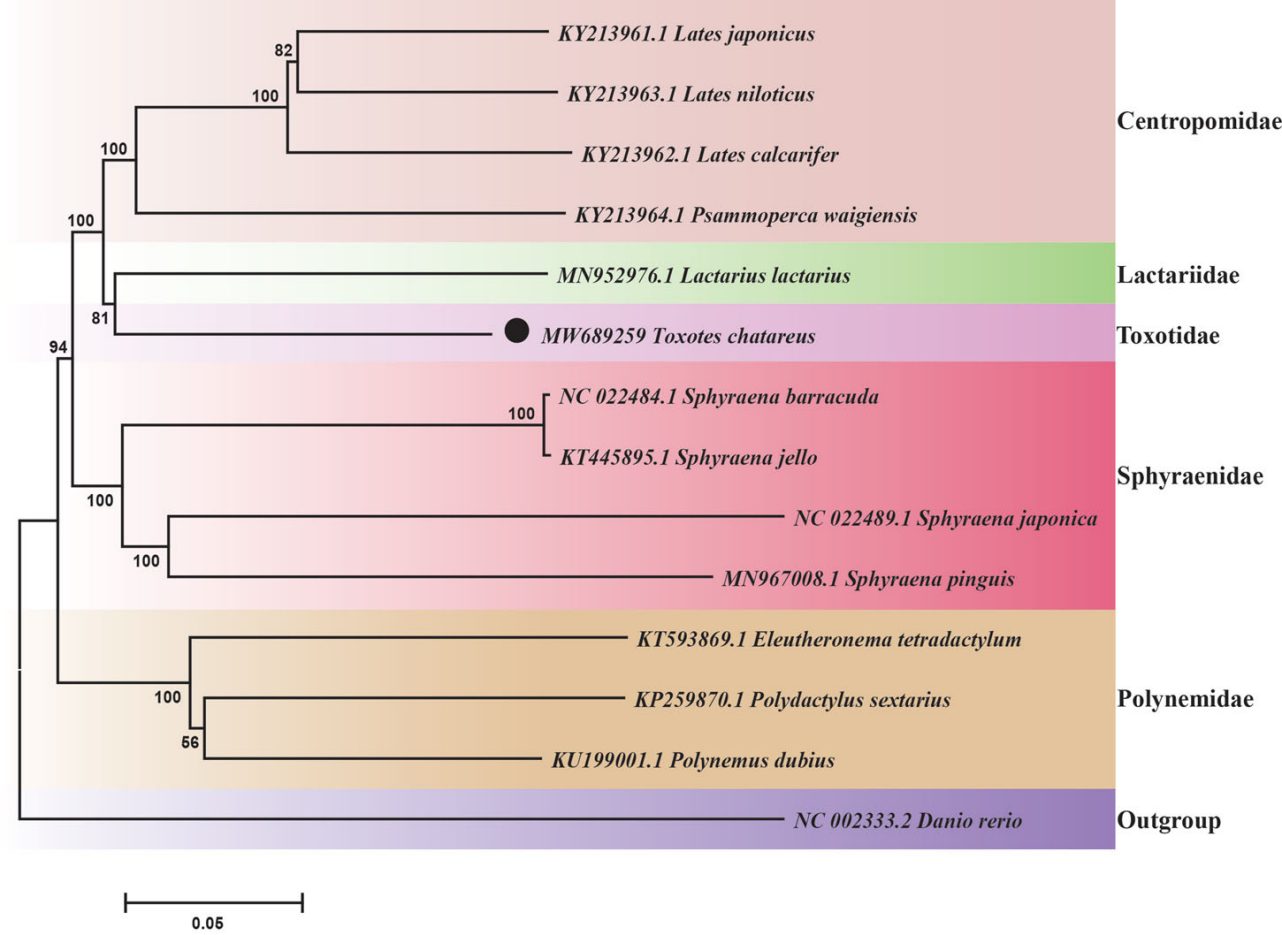
Neighbor-Joining tree of 13 Carangaria and one outgroup (Danio rerio) species based on the complete mitochondrial genome. The number at each node represents the bootstrap probability. The dark spot indicates the studied species.

## Data Availability

The mitochondrial genome sequence data that support the findings of this study are openly available in GenBank of NCBI at https://www.ncbi.nlm.nih.gov under the accession number MW689259. The associated BioProject, SRA, and Bio-Sample numbers are PRJNA592745, SRR11793814, and SAMN13439313, respectively.

## References

[CIT0001] Allen GR. 1978. A review of the archerfishes (family Toxotidae). Rec West Aust Mus. 6.

[CIT0002] Allen GR. 2001. Toxotidae-archer fishes. In: Carpenter KE, Niem VH, editors. FAO species identification guide for fishery purposes. The living marine resources of the Western Central Pacific. Rome: FAO; p. 3212–3215.

[CIT0003] Allen GR. 2004. *Toxotes kimberleyensis*, a new species of archerfish (Pisces: Toxotidae) from fresh waters of Western Australia. Rec Aust Mus. 56(2):225–230.

[CIT0004] Bernt M, Donath A, J¨Uhling F, Externbrink F, Florentz C, Fritzsch G, P¨Utz J, Middendorf M, Stadler P. 2013. MITOS: improved de novo metazoan mitochondrial genome annotation. Mol Phylogenet Evol. 69(2):313–319. 22982435 10.1016/j.ympev.2012.08.023

[CIT0005] Chan PP, Lowe TM. 2019. tRNAscan-SE: searching for tRNA genes in genomic sequences. Methods Mol Biol. 1962:1–14. 31020551 10.1007/978-1-4939-9173-0_1 PMC6768409

[CIT0006] Elshoud GCA, Koomen P. 1985. A biomechanical analysis of spitting in archer fishes (Pisces, Perciformes, Toxidae). Zoomorphology. 105(4):240–252.

[CIT0007] Froese R, Pauly D. 2021. World Wide Web electronic publication. FishBase, www.fishbase.org.

[CIT0008] Gan H, Takahashi H, Hammer M, Tan M, Lee Y, Voss J, Austin C. 2017. Mitochondrial genomes and phylogenetic relationships of *Lates japonicus*, *Lates niloticus*, and *Psammoperca waigiensis* (Perciformes: Latidae). Mitochondrial DNA B Resour. 2(1):73–75. 33473721 10.1080/23802359.2017.1285206 PMC7800192

[CIT0009] Hall TA. 1999. BioEdit: a user-friendly biological sequence alignment editor and analysis program for Windows 95/98/NT. Nucl Acids Symp Ser. 41:95–98.

[CIT0010] Karoubi N, Segev R, Wullimann M. 2016. The brain of the archerfish *Toxotes chatareus*: a nissl-based neuroanatomical atlas and catecholaminergic/cholinergic systems. Front Neuroanat. 10:106. 27891081 10.3389/fnana.2016.00106 PMC5104738

[CIT0011] Kumar S, Stecher G, Tamura K. 2016. MEGA7: molecular evolutionary genetics analysis version 7.0 for bigger datasets. Mol Biol Evol. 33(7):1870–1874. 27004904 10.1093/molbev/msw054 PMC8210823

[CIT0012] Lü Z, Gong L, Ren Y, Chen Y, Wang Z, Liu L, Li H, Chen X, Li Z, Luo H, et al. 2021. Large-scale sequencing of flatfish genomes provides insights into the polyphyletic origin of their specialized body plan. Nat Genet. 53(5):742–751. 33875864 10.1038/s41588-021-00836-9 PMC8110480

[CIT0013] Lv H, Cheng Q, Pang J, Zhang H. 2016. The complete mitochondrial genome sequence of *Sphyraena jello* (Perciformes: Sphyraenidae) and its phylogenetic position. Mitochondrial DNA A DNA Mapp Seq Anal. 27(6):4570–4571. 26641714 10.3109/19401736.2015.1101568

[CIT0014] Meng G, Li Y, Yang C, Liu S. 2019. MitoZ: a toolkit for animal mitochondrial genome assembly, annotation and visualization. Nucleic Acids Res. 47(11):e63. 30864657 10.1093/nar/gkz173 PMC6582343

[CIT0015] Schoch CL, Ciufo S, Domrachev M, Hotton CL, Kannan S, Khovanskaya R, Leipe D, Mcveigh R, O’Neill K, Robbertse B, et al. 2020. NCBI Taxonomy: a comprehensive update on curation, resources and tools. Database (Oxford). 2020. 10.1093/database/baaa062 PMC7408187 32761142

[CIT0016] Simon KD, Bakar Y, Mazlan AG, Zaidi CC, Samat A, Arshad A, Temple SE, Brown-Peterson NJ. 2012. Aspects of the reproductive biology of two archer fishes *Toxotes chatareus*, (Hamilton 1822) and *Toxotes jaculatrix* (Pallas 1767). Environ Biol Fish. 93(4):491–503.

[CIT0017] Simon KD, Bakar Y, Samat A, Zaidi CC, Aziz A, Mazlan AG. 2009. Population growth, trophic level, and reproductive biology of two congeneric archer fishes (*Toxotes chatareus*, Hamilton 1822 and *Toxotes jaculatrix*, Pallas 1767) inhabiting Malaysian coastal waters. J Zhejiang Univ Sci B. 10(12):902–911. 19946954 10.1631/jzus.B0920173 PMC2789525

[CIT0018] Simon K, Bakar Y, Temple S, Mazlan A. 2010. Morphometric and meristic variation in two congeneric archer fishes *Toxotes chatareus* (Hamilton 1822) and *Toxotes jaculatrix* (Pallas 1767) inhabiting Malaysian coastal waters. J Zhejiang Univ Sci B. 11(11):871–879. 21043056 10.1631/jzus.B1000054 PMC2970897

[CIT0019] Tabassum N, Park W, Baek H, Je J, Kim H. 2020. Characterization of the complete mitochondrial genome of brown barracuda, *Sphyraena pinguis* (Perciformes: Sphyraenidae). Mitochondrial DNA B Resour. 5(3):3042–3043. 33458050 10.1080/23802359.2020.1797588 PMC7782257

[CIT0020] Temple SE. 2007. Effect of salinity on the refractive index of water: considerations for archer fish aerial vision. Journal of Fish Biology. 70(5):1626–1629.

[CIT0021] Temple S, Hart NS, Marshall NJ, Collin SP. 2010. A spitting image: specializations in archerfish eyes for vision at the interface between air and water. Proc Biol Sci. 277:2607–2615. 20392734 10.1098/rspb.2010.0345 PMC2982040

[CIT0022] Temple SE, Manietta D, Fau-Collin SP, Collin SP. 2013. A comparison of behavioural (Landolt C) and anatomical estimates of visual acuity in archerfish (*Toxotes chatareus*). Vision Res. 83:1–8. 23466473 10.1016/j.visres.2013.02.014

[CIT0023] Thompson JD, Higgins DG, Gibson TJ. 1994. CLUSTAL W: improving the sensitivity of progressive multiple sequence alignment through sequence weighting, position-specific gap penalties and weight matrix choice. Nucleic Acids Res. 22(22):4673–4680. 7984417 10.1093/nar/22.22.4673 PMC308517

[CIT0024] Timmermans P. 2001. Prey catching in the archer fish: angles and probability of hitting an aerial target. Behav Processes. 55(2):93–105. 11470501 10.1016/s0376-6357(01)00172-3

[CIT0025] Wang C, Qiu J, Peng X, Ai W, Huang X, Liu W, Chen S. 2016. The complete mitochondrial genome of *Polydactylus sextarius* (Teleostei, Mugiliformes). Mitochondrial DNA A DNA Mapp Seq Anal. 27(5):3344–3345. 25714147 10.3109/19401736.2015.1018214

[CIT0026] Yang M, Li P, Qin Q, Zhu K. 2019. The complete mitochondrial genome of false trevally *Lactarius Lactarius* (Bloch and Schneider, 1801). Mitochondrial DNA B Resour. 5(1):87–89. 33366435 10.1080/23802359.2019.1698335 PMC7720941

